# Spatio-Temporal Patterns of Beaked Whale Echolocation Signals in the North Pacific

**DOI:** 10.1371/journal.pone.0086072

**Published:** 2014-01-22

**Authors:** Simone Baumann-Pickering, Marie A. Roch, Robert L. Brownell Jr, Anne E. Simonis, Mark A. McDonald, Alba Solsona-Berga, Erin M. Oleson, Sean M. Wiggins, John A. Hildebrand

**Affiliations:** 1 Scripps Institution of Oceanography, University of California, San Diego, La Jolla, California, United States of America; 2 Department of Computer Science, San Diego State University, San Diego, California, United States of America; 3 Southwest Fisheries Science Center, National Oceanic and Atmospheric Administration, Pacific Grove, California, United States of America; 4 WhaleAcoustics, Bellvue, Colorado, United States of America; 5 Universitat de Barcelona, Barcelona, Spain; 6 Pacific Islands Fisheries Science Center, National Oceanic and Atmospheric Administration, Honolulu, Hawaii, United States of America; Texas A&M University-Corpus Christi, United States of America

## Abstract

At least ten species of beaked whales inhabit the North Pacific, but little is known about their abundance, ecology, and behavior, as they are elusive and difficult to distinguish visually at sea. Six of these species produce known species-specific frequency modulated (FM) echolocation pulses: Baird’s, Blainville’s, Cuvier’s, Deraniyagala’s, Longman’s, and Stejneger’s beaked whales. Additionally, one described FM pulse (BWC) from Cross Seamount, Hawai’i, and three unknown FM pulse types (BW40, BW43, BW70) have been identified from almost 11 cumulative years of autonomous recordings at 24 sites throughout the North Pacific. Most sites had a dominant FM pulse type with other types being either absent or limited. There was not a strong seasonal influence on the occurrence of these signals at any site, but longer time series may reveal smaller, consistent fluctuations. Only the species producing BWC signals, detected throughout the Pacific Islands region, consistently showed a diel cycle with nocturnal foraging. By comparing stranding and sighting information with acoustic findings, we hypothesize that BWC signals are produced by ginkgo-toothed beaked whales. BW43 signal encounters were restricted to Southern California and may be produced by Perrin’s beaked whale, known only from Californian waters. BW70 signals were detected in the southern Gulf of California, which is prime habitat for Pygmy beaked whales. Hubb’s beaked whale may have produced the BW40 signals encountered off central and southern California; however, these signals were also recorded off Pearl and Hermes Reef and Wake Atoll, which are well south of their known range.

## Introduction

The North Pacific is inhabited by at least ten species of beaked whales. These are: Baird’s (*Berardius bairdii,* Bb), Cuvier’s (*Ziphius cavirostris,* Zc), Longman’s (*Indopacetus pacificus,* Ip), Blainville’s (*Mesoplodon densirostris,* Md), Stejneger’s (*M. stejnegeri,* Ms), Hubb’s (*M. carlhubbsi,* Mc), Perrin’s (*M. perrini,* Mpe), Ginkgo-toothed (*M. ginkgodens,* Mg) and Pygmy beaked whale (*M. peruvianus,* Mpu) [1]. The tenth species is the Deraniyagala’s beaked whale, *M. hotaula* (Mh) [2], which recently has been resurrected as a separate species from the morphologically similar *M. ginkgodens* [3]. Information on the abundance, distribution, and community structure of all these species is limited because of their highly elusive behavior and the small numbers of strandings and visual sightings. They are all deep-diving odontocetes that undergo long foraging dives with short surface intervals [4].

**Figure 1:**
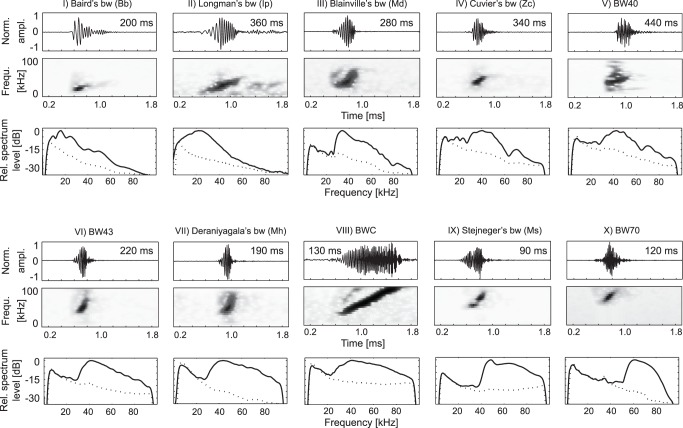
Overview of frequency-modulated (FM) upsweep pulses from known (I–IV, VII, IX) and unknown sources (V, VI, VIII, X,). Each FM pulse type is shown with an example pulse time series (top) and spectrogram (middle, Hann-windowed 2 ms, 40-point DFT, 97% overlap), as well as a mean spectra (bottom, solid line) over all FM pulses from several acoustic encounters and mean noise (dotted line) extracted before each pulse. Inter-pulse interval (IPI) is specified in ms (value in time series plot).

In recent years, advances have been made in acoustically identifying beaked whales by their echolocation signals. These signals are mostly frequency-modulated (FM) upsweep pulses, which appear to be species-specific and distinguishable by their spectral and temporal features [5] (Figure 1). From the North Pacific, we are able to identify, based on recordings obtained with concurrent visual observations, four FM pulses made by Baird’s, Blainville’s, Cuvier’s, and Longman’s beaked whale [6]–[12]. The species visually and acoustically observed at Palmyra Atoll is likely Deraniyagala’s beaked whale [13]. Baumann-Pickering et al. [14] associated FM pulses recorded in the Aleutian Islands with autonomous passive acoustic recorders as belonging to Stejneger’s beaked whales. This association was based on two factors. Two of the three FM signal types occurring in the region, albeit infrequently, correspond well to descriptions of Baird’s and Cuvier’s beaked whales [8], [11]. Stejneger’s is the only other beaked whale known to inhabit this area suggesting that it is also the source of the most commonly detected FM pulse near the Aleutian Islands.

One distinct FM pulse of unknown origin was described for a yearlong recording on top of Cross Seamount (BWC), southwest of the Island of Hawai’i, likely produced by a beaked whale [15]. Additionally, three FM pulse types (BW40, BW43, BW70, named by their peak frequency), similar to those being produced by beaked whales, have been identified on autonomous acoustic recordings throughout the North Pacific [5]. These signals have distinct spectral and temporal features used for discrimination (Figure 1).

Passive acoustics have been used to distinguish stocks as well as describe their geographic ranges for a number of marine mammal species such as killer (*Orcinus orca)*, blue (*Balaenoptera musculus)*, and fin (*Balaenoptera physalus*) whales [16]–[18]. In this work, we examine the geospatial characteristics of North Pacific beaked whale species. We consider the distribution of unknown FM pulse types along with the well-described ones in the context of known distributions for beaked whales from stranding and sighting data. We describe the spatio-temporal distribution and relative abundance of North Pacific beaked whales based on the acoustic detections of FM pulses on long-term autonomous acoustic recorders from 24 sites over the years 2005 to 2012.

## Materials and Methods

### Ethics Statement

High-frequency Acoustic Recording Packages were deployed near Palmyra Atoll under U.S. Fish & Wildlife Service Special Use Permit 12533, at Pearl & Hermes Reef under Papahanaumokuakea Marine National Monument permit PMNM-2008-020, in the Gulf of California under La Secretaria de Relaciones Exteriores permit DAN-01342 and DAN-00415, and off the coast of Washington, U.S., under Olympic Coast National Marine Sanctuary permit OCNMS-2006-003 and OCNMS-2010-010. All other deployment sites did not need permitting and fieldwork did not involve endangered or protected species since the recordings were made passively.

### Data Collection

**Figure 2:**
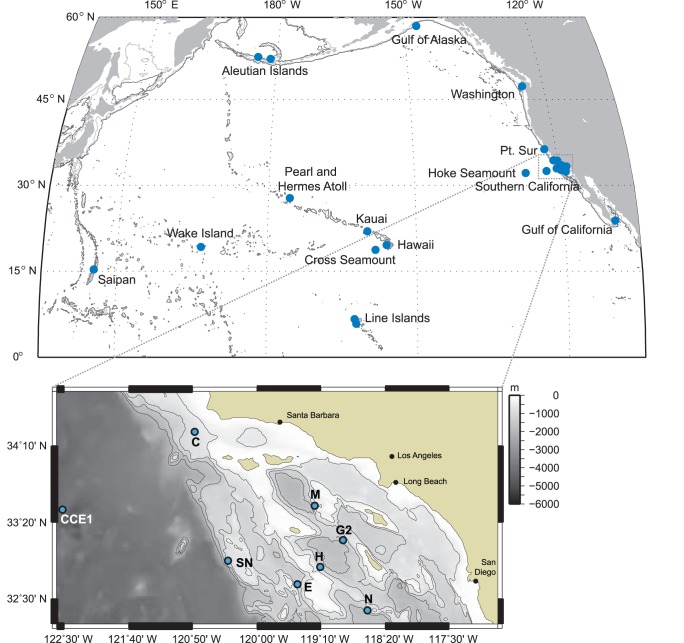
Locations of HARP recording sites (circles) across the North Pacific (top) and within the Southern California Bight (bottom).

Acoustic recordings were collected with autonomous High-frequency Acoustic Recording Packages (HARPs) [19] from 24 sites in the North Pacific mainly along the west coast of North America and the Pacific Islands region (Table 1 Table 1, Figure 2). Within the eastern North Pacific, recording effort occurred at several sites along the west coast of the United States including one site in the Gulf of Alaska, two sites off the coast of Washington, one site offshore of central California, several sites throughout the Southern California Bight and offshore on Hoke Seamount (west of San Diego, California), and one site near the tip of the Baja California Peninsula in the Gulf of California. The Southern California Bight had the highest effort with seven sites at various bathymetric features within the Bight. Within the central North Pacific, recording effort occurred at two sites in the Aleutian Islands, and at several more tropical sites within the Main and Northwestern Hawaiian Islands (Hawai’i, Kaua’i, Cross Seamount, and Pearl and Hermes Reef), in the Northern Line Islands (Palmyra Atoll and Kingman Reef), and Wake Atoll. A single site has been monitored within the western North Pacific near Saipan in the Northern Mariana Islands. HARPs at the different sites had a variety of recording durations from several weeks to over one year and recording schedules ranging from continuous to 5 minutes of recording every 40 minutes. Sites were often maintained over several deployment missions resulting in a total of 19 years of analyzed deployment time (approximately 11 years of actual recording time, accounting for recording schedule when HARP was deployed but not continuously recording) over the period 2005 to 2012. All recorders were deployed to seafloor depths between 700 and 1400 m, except one at 100 m. HARPs were bottom-mounted, either in a seafloor-packaged configuration or as a mooring with the hydrophone at about 10 to 30 m, respectively, above the seafloor. All HARPs were set to a sampling frequency of 200 kHz with 16-bit quantization. The recorders were equipped with an omni-directional sensor (ITC-1042, International Transducer Corporation, Santa Barbara, CA), which had an approximately flat (±2 dB) hydrophone sensitivity from 10 Hz to 100 kHz of –200 dB re V/µPa. The sensor was connected to a custom-built preamplifier board and bandpass filter. The preamplifiers were designed to flatten the frequency response of the ambient ocean noise, which provided greater gain at higher frequencies where ambient noise levels are lower and sound attenuation is higher [19]. The calibrated system response was corrected for during analysis.

**Table 1:** HARP deployment details with recording start and end dates spanning multiple deployments, geographic locations and depths.

Project	Site	RecordingStart	RecordingEnd	Longitude		Latitude	Depth(m)	RecordingSchedule	# Deploy	Recording Days
**North East Pacific**										
Aleutian Islands	Kiska	03-Jun-10	20-Jul-10	178° 31.240′′ E		52° 19.007′′ N	1100	cont.	1	48
Aleutian Islands	Buldir	27-Aug-10	26-May-11	175° 37.990′′ E		52° 38.000′′ N	800	cont.	1	272
Gulf of Alaska	CB	13-Jul-11	19-Feb-12	148° 04.129′′ W		58° 38.741′′ N	1000	cont.	1	221
Washington	Cape Elizabeth	17-Jun-08	06-Nov-11	124° 43.256′′ W		47° 21.117′′ N	100	1/7 or cont.	2	527
Washington	Quinault Canyon	27-Jan-11	07-Oct-11	125° 21.203′′ W		47° 30.003′′ N	1400	cont.	1	253
Point Sur	Point Sur	03-Oct-06	16-Jan-07	122° 23.628′′ W		36° 17.946′′ N	1392	1/3	1	105
Hoke Seamount	Hoke	15-Sep-08	06-Jun-09	126° 54.580′′ W		32° 06.370′′ N	770	1/7	1	265
Gulf of California	Punta Pescadero	27-Nov-05	05-Jun-07	109°37.668′′ W		23° 49.645′′ N	750	1/5 or cont.	3	401
**Southern California (SOCAL)**										
SOCAL	C	12-Mar-09	05-May-09	120° 48.367′′ W	34° 18.885′′ N		800	cont.	1	54
SOCAL	E	03-Sep-06	12-Jul-09	119° 28.389′′ W	32° 39.379′′ N		1300	cont.	6	322
SOCAL	G2	13-Jan-09	04-Mar-09	118° 52.815′′ W	33° 08.407′′ N		1150	cont.	1	51
SOCAL	H	05-Jun-08	03-Jan-09	119° 10.624′′ W	32° 50.823′′ N		1000	cont.	6	278
SOCAL	M	13-Jan-09	02-Oct-11	119° 14.875′′ W	33° 30.887′′ N		950	cont.	10	804
SOCAL	N	14-Jan-09	23-Sep-11	118° 33.803′′ W	32° 22.189′′ N		1300	cont.	8	597
SOCAL	SN	19-May-09	02-Jun-10	120° 22.544′′ W	32° 54.913′′ N		1100	1/7	1	379
**Pacific Islands**										
Main Hawaiian Islands	Hawai’i	23-Apr-09	16-Jun-10	156° 00.930′′ W	19° 34.889′′ N		600	1/3, 1/5, 3/7or cont.	4	294
Main Hawaiian Islands	Kaua’i	08-Oct-09	20-Aug-10	159° 53.383′′ W	21° 57.224′′ N		700	1/4 or cont.	2	294
Cross Seamount	Cross	20-Nov-05	11-May-06	158° 15.221′′ W	18° 43.343′′ N		396/398	1/5	1	172
NW Hawaiian Islands	Pearl & Hermes Reef	20-Oct-09	17-Sep-10	175° 37.946′′ W	27° 43.620′′ N		750	1/4 or cont.	2	325
Northern Line Islands	Palmyra Atoll WT	19-Oct-06	02-Apr-09	162° 09.385′′ W	05° 51.777′′ N		600	1/4	4	595
Northern Line Islands	Palmyra Atoll NS	02-Jun-09	25-Aug-10	162° 02.224′′ W	05° 53.690′′ N		700/1100	1/4 or cont.	3	230
Northern Line Islands	Kingman Reef	20-Oct-11	11-Mar-12	162° 17.539′′ W	06° 21.908′′ N		850	cont.	1	144
Pacific Islands	Wake Atoll	31-Jan-10	04-May-10	166° 41.000′′ E	19° 13.000′′ N		800	1/2	1	94
Northern Mariana Islands	Saipan	05-Mar-10	25-Aug-10	145° 27.542′′ E	15° 18.998′′ N		700	1/8	1	174
									**Total**	**19 years**

Depth values indicate approximate depth of recorder and hydrophone near the seafloor, value in parentheses indicates seafloor depth when the hydrophone was positioned much shallower than the seafloor, multiple values indicate different depths over different deployments near the same site. Recording schedule was either continuous (cont.) or on a fractional schedule with the number of 5 minute recording periods over the total number of 5 minute periods per cycle. Recording days are the sum of recorded days of all deployments, not accounting for recording schedule.

### Signal Detection and Classification

Signal processing was performed using the MATLAB (Mathworks, Natick, MA) based custom software program *Triton* [19] and other MATLAB custom routines. Trained analysts (SBP, AES, ASB, MAM) manually identified beaked whale-like frequency-modulated (FM) echolocation pulses in the HARP data. Datasets were divided between analysts and missed detections due to possible differences in analyst performance were not quantified. These signals had, in comparison to known delphinid clicks, longer durations, a stable inter-pulse interval (IPI), and an upswept frequency. Long-term spectral averages (LTSAs) were calculated for visual analysis of the long-term recordings. LTSAs are long-term spectrograms with each time segment consisting of an average of 500 spectra, which were created using the Welch algorithm [20]. The averages were formed from the power spectral densities of non-overlapped 10 ms Hann-windowed frames. The resulting long-term spectrograms have a resolution of 100 Hz in frequency and 5 seconds in time. When echolocation signals were notable in the LTSA, the sequence was inspected more closely. A number of parameters were used to evaluate each signal’s characteristics. Time series of 5 s lengths showed IPI, time series of 3 ms lengths was used to display the shape of the waveform, and spectrograms of Hann-windowed 3 ms segments (60-points DFT, 98% overlap) revealed the presence of FM pulses. Start and end times of acoustic encounters were noted if beaked whale like FM pulses were identified. Analysts initially labeled these acoustic encounters as (1) having been produced by one of the species whose echolocation signals are well known, (2) one of the groups of echolocation signal categories whose origin has not yet been determined, or (3) as unidentifiable with beaked whale echolocation signal characteristics.

All presumed beaked whale acoustic encounters were reviewed in several additional analysis steps. Individual echolocation signals were automatically detected using a two-step approach computer algorithm during time periods when FM pulses were manually detected [21]. The individual FM pulse detections were digitally filtered with a 10-pole Butterworth band-pass filter with a pass-band between 5 kHz and 95 kHz. Filtering was done on 800 sample points centered on the echolocation signal. Spectra of each detected signal were calculated using 2.56 ms (512 samples) of Hann-windowed data centered on the signal. Peak frequency was determined as the spectral frequency with the highest amplitude. FM pulse duration was derived from the detector output and IPIs were calculated from the start of an FM pulse to the start of the previous one. All detected echolocation signals, independent of distance and orientation of the recorded animal with respect to the recorder, were included in the analysis. A software tool displayed for each acoustic encounter histograms of peak frequency and IPI, mean spectra with mean noise preceding each click, and concatenated spectra [5]. This signal discrimination tool overlaid the mean spectra of the acoustic detection against spectral templates of all beaked whale FM pulse types. The analyst optionally browsed through plots of individual time series and spectrograms (2 ms Hann-windowed data, 40-point DFT, 97% overlap) of echolocation signals detected within the acoustic encounter, sorted by peak-to-peak received level displaying high quality signals first. This led to a final judgment about the label for each acoustic encounter and the analyst submitted a decision. In case the acoustic encounter was not grouped to one of the ten FM pulse types, based on low quality of the acoustic encounter, very few FM pulse detections, or based on spectral and temporal characteristics that were atypical of our observed FM pulse types, the acoustic encounter was labeled as a probable unidentified beaked whale” (UBW), being an inhomogeneous group, likely comprised of a variety of FM pulse types and were not used in the analysis. The analyst’s decisions for the rare signal types and a subset of frequent types were reviewed by SBP to assure consistency with decisions.

### Statistical Analysis

Analysis of the spatial distribution of each FM pulse type and the relative occurrence of FM pulse types occurring at each site provides insight into the geographic range of each FM pulse type and relative probability of encounters for a given area. For acoustic encounters of beaked whale species with known signal types this may refine the spatio-temporal knowledge of these species. For species whose signal types are currently unknown but whose geographic range is identified, a geographic overlap with unassociated signal types might provide information leading to which species produces which signal type.

Relative occurrence of beaked whale echolocation signals was analyzed with respect to the proportions of FM pulse types at specific sites and to FM pulse type distribution across sites. Both analyses examined presence/absence of FM pulse types on a daily basis. The decision to use daily presence was based upon varying duty cycles that affect the probability of detection for any given site. Examination of a longer period increases the probability that a whale utilizing the habitat is detected and reduces compositional biases that might occur due to differences in dive and echolocation behavior. Further normalization would require an estimate of the probability of detection.

Per site analysis highlights the relative presence of FM pulse types. The number of days with detections for each species was summed, and the percentage of days attributed to each pulse type is reported. An overall relative presence is also computed to assess how often the habitat within the site’s detection area is used. This was defined as the percentage of recording days for which there was a detection of any FM pulse type. A relative detection effort was defined as the proportion of the effort [0,1] relative to the site with the greatest deployment duration in days.

Geographic analysis of FM pulse types required normalization for the effort at each site. The number of daily encounters for a specific pulse type was divided by the number of physical days (ignoring duty cycle as discussed earlier) that instruments were deployed and the relative distribution across sites is reported.

For seasonal analysis, the sum of acoustic encounter durations was used as a measure. This allows detecting smaller fluctuations in abundance, which may remain unnoticed looking at daily presence/absence data. Encounter durations were adjusted for recording effort on a per site, per species basis to permit pooling of data across deployments with different duty cycles. Monthly acoustic encounter durations were scaled [0,1] to the greatest sum per site, to permit comparison of presence between sites. For diel patterns, hourly presence and absence counts were computed by FM pulse type over all sites and on a per site basis.

## Results

### Relative Presence

The highest relative daily presence for beaked whale signals occurred at Kingman Reef (Table 2, relative presence) followed closely by Perl & Hermes Reef, Wake Atoll and Southern California site E. High relative presence (60%–80%) was detected at: Southern California G2 and H as well as Gulf of Alaska CB. Moderate relative presence (40%–60%) was found at: Southern California sites C and N, the North shore (NS) of Palmyra Atoll, and Cross Seamount off the Hawaiian Islands. Lower relative presence (20–40%) occurred at: Hoke Seamount, Point Sur offshore of Central California, sites M and SN offshore of Southern California, Saipan, Quinault Canyon offshore of Washington, Aleutian Islands Kiska, and the Hawaiian sites of Hawai’i and Kaua’i. Finally, beaked whale signals were encountered on less than 10% of the days at: Aleutian Islands Buldir, Gulf of California Punta Pescadero, the Western terrace (WT) of Palmyra Atoll, and Cape Elizabeth offshore of Washington.

**Table 2:** Relative distribution of daily presence of acoustic encounters for all FM pulse types by site, normalized for days of effort.

Project	Site	Relative presence %	Bb	Md	Mh	Ms	Zc	BW40	BW43	BW70	BWC
**North East Pacific**											
Aleutian Islands	Kiska	31				93	7				
Aleutian Islands	Buldir	6	6			94					
Gulf of Alaska	CB	67	35			64	2				
Washington	Cape Elizabeth	*	100								
Washington	Quinault Canyon	34	26	1		73					
Point Sur	Point Sur	36					95	5			
Hoke Seamount	Hoke	38					97		3		
Gulf of California	Punta Pescadero	2								100	
**Southern California (SOCAL)**											
SOCAL	C	46	96					4			
SOCAL	E	81	5				93	1	1		
SOCAL	G2	74					100				
SOCAL	H	70					99	1			
SOCAL	M	23	6			2	91	1			
SOCAL	N	55	5				93	1	1		
SOCAL	SN	35	4				92	3	1		
**Pacific Islands**											
Main Hawaiian Islands	Hawai’i	22		90			6				4
Main Hawaiian Islands	Kaua’i	20		68							32
Cross Seamount	Cross	47									100
NW Hawaiian Islands	Pearl and Hermes Reef	88		59			38	1			3
Northern Line Islands	Kingman Reef	100		6	78		15				2
Northern Line Islands	Palmyra Atoll NS	49			99						1
Northern Line Islands	Palmyra Atoll WT	5		3	90		6				
Pacific Islands	Wake Atoll	88					87	5			7
Northern Mariana Islands	Saipan	34		63			9				28

Relative presence is reported by the percentage of recording days with detections. Star (*) indicates a value with less than 1%. Grey shaded area shows no encounters of a type at a site.

**Figure 3:**
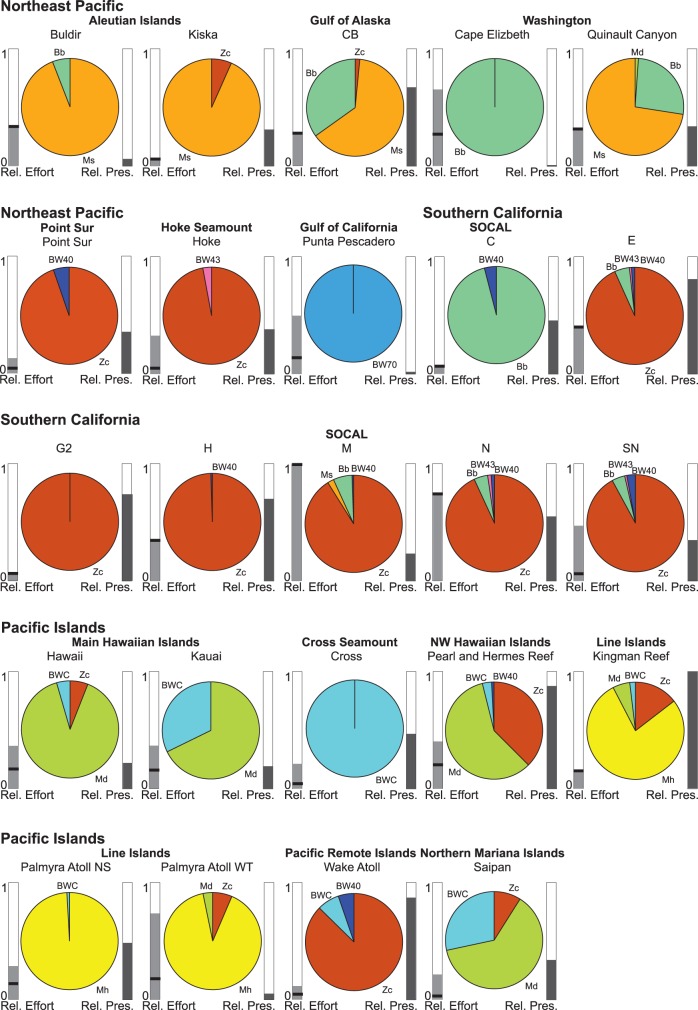
Relative daily occurrence of FM pulse types by site (pie chart, see Table 2). For each pie chart, the relative detection effort (number of days recorded) is displayed on the left, and the relative presence on the right. Site M in the Southern California Bight had the highest effort (relative effort of 1) and Kingman Reef had the highest percentage of days with acoustic encounters (relative presence of 1). A dark line through the relative effort is indicative of duty cycle and reflects the amount of continuous effort.

A site-specific analysis, looking at the presence of encounters over a 24-hour period per species and site, revealed that up to four pulse types occurred at each site (mean = 2.6, SD = 1.0). Each site had one highly dominant FM pulse type. The dominant type accounted for a mean of 88% (SD = 13%) of the days with detections for each FM pulse type across all sites (Table 2). Zc type signals dominated all of the SOCAL sites except SOCAL C, which had highest acoustic encounter rates for Bb signals. Zc signals were also dominant at Point Sur, Hoke Seamount, and Wake Atoll. Md signals were the prevailing ones at Hawai’i, Kaua’i, Pearl and Hermes Reef, and Saipan. Ms FM pulses were most often encountered on recordings of the Aleutian Islands sites, Gulf of Alaska, and the deep Washington site Quinault Canyon. Mh signals were dominant at Kingman Reef and both Palmyra Atoll sites. BWC signals were the only FM pulse type acoustically encountered at Cross Seamount (southwest of the Island of Hawai’i). BW70 signals were the only FM pulse type at the Gulf of California site (Figure 3, Table 2). The BW40 and BW43 signal types were less frequently observed, with neither call type playing a dominant role at any of the sites. The Ip signal type was encountered only a few times, likely with a larger number of missed detections, precluding it from being used in this study.

For a signal-specific analysis, the same daily presence counts were reorganized to show the distribution of each FM pulse type by site after accounting for the number of days of recording effort (Table 3). This representation highlights the geographic range of detections of each call type. Zc FM pulses were the signal type with the broadest distribution over all areas of the monitored North Pacific, with frequent encounters at SOCAL E, G2, H, N, Pearl and Hermes Reef, and Wake Atoll (9 to 15% of daily FM pulse detections for each site, Table 3), to a lesser degree at the Aleutian Islands site Kiska, in the Gulf of Alaska, central and offshore California, SOCAL M, N, Hawai’i, Northern Line Islands, and Saipan (<1–7%, Table 3). Acoustic encounters of Md signals were frequently found, but were restricted to the Pacific Islands region with the exception of one encounter offshore of Washington State. Mh signals were only recorded at Northern Line Islands sites of Palmyra Atoll and Kingman Reef. Different Mh encounter rates at the two Palmyra sites (north shore 32% and western terrace 3%) may be indicative of preferential habitat usage. BWC signals were encountered acoustically in all sites in the Pacific Islands except Palmyra WT, with the most relative presence during 59% of effort days at Cross Seamount, followed by 13% at Saipan. Ms and Bb FM pulse types were more common at the cooler, northern sites, such as the Aleutian Islands, Gulf of Alaska, and Washington, as well as various SOCAL sites. Ms signals were more frequently encountered near the Aleutians, Gulf of Alaska, and offshore of Washington. Bb signal type acoustic encounters were highest at SOCAL C (46%). The BW70 FM pulse was only found on the Gulf of California recordings. The BW43 FM pulse was most often detected at Hoke Seamount, comprising 42% of its encountered days, as well as at the sites closer to the shelf break at SOCAL E, N, and SN. FM pulse type BW40 was encountered at Wake Atoll and Pearl and Hermes Reef, as well as off the coast of central and southern California.

**Table 3:** Percentage of number of days with detections per FM pulse type over all sites.

Signal type		North East Pacific								Southern California (SOCAL)							Pacific Islands								
	Number of acoustic encounters (not accounting for effort)	Aleutian Islands	Aleutian Islands	Gulf of Alaska	Washington	Washington	Point Sur	Hoke Seamount	Gulf of California	SOCAL	SOCAL	SOCAL	SOCAL	SOCAL	SOCAL	SOCAL	Main Hawaiian Islands		Cross Seamount	NW Hawaiian Islands	Northern Line Islands	Northern Line Islands	Northern Line Islands	Pacific Islands	Northern Mariana Islands
		Kiska	Buldir	CB	Cape Elizabeth	Quinault Canyon	Point Sur	Hoke	Punta Pescadero	C	E	G2	H	M	N	SN	Hawai’i	Kaua’i	Cross	Pearl and Hermes Reef	Kingman Reef	NS	WT	Wake Atoll	Saipan
Bb	248		*	32	*	10				46	5			2	3	2									
Md	1736					*											13	9		57	5		*		16
Mh	1546																				65	32	3		
Ms	437	25	5	48		22								*											
Zc	4478	*		*			6	7			14	13	12	4	9	6	*			10	3		*	15	*
BW40	25						14			14	7		3	*	5	8				9				40	
BW43	10							42			23				25	10									
BW70	8								100																
BWC	258																1	8	59	6	3	*		9	13

Star indicates value with less than 1%. Grey shaded area shows no encounters of a type.

### Seasonality

Good yearly coverage was available for SOCAL, particularly sites M and N with 3 years of nearly continuous data, Palmyra Atoll, and the Gulf of California (Table 1). Multi-year coverage was also achieved by looking at regional rather than site-by-site scale. The paucity of acoustic encounters severely hampered the ability to make seasonal inferences for most FM pulse types. Amongst the signal types that occurred infrequently, BW43 and BW70 occurred at a number of SOCAL sites and at a Gulf of California site, respectively, in various months throughout the year. Bb and Mh signals were recorded in the North-Eastern Pacific and the Northern Line Islands sites, respectively, throughout the recording period without seasonal pattern.

**Figure 4:**
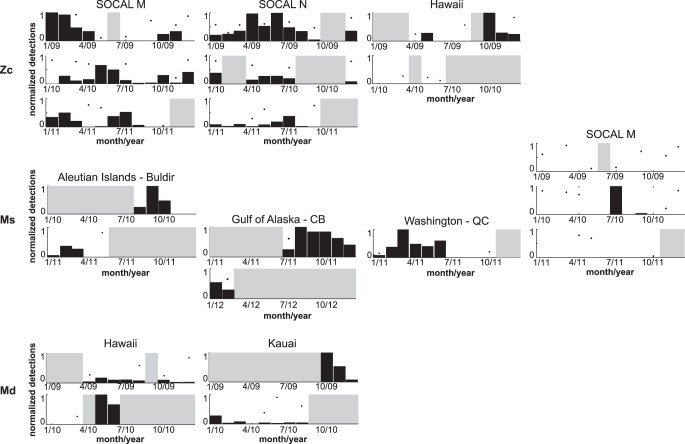
Seasonality of *Z. cavirostris* (Zc, top panel) at sites SOCAL M, N and Hawai’i, of *M. stejnegeri* (Ms, middle panel) at sites Aleutian Islands Buldir, Gulf of Alaska CB, Washington Quinault Canyon, and SOCAL M, and of *M. densirostris* (Md, bottom panel) at sites Hawai’i and Kaua’i. A point indicates partial monthly recording effort and data in that month was adjusted for reduced effort. Grey shaded areas show no monthly effort.

However, one seasonal pattern and some shorter-term variations were noteworthy (Figure 4). Zc signals showed a higher occurrence at SOCAL M and N during the summer in three consecutive monitoring years, except at site M in 2009 when winter months had the highest acoustic encounter rates. In a little over one year of recording at the Hawai’i site, Zc signals were relatively rare (Table 3), with higher numbers of encounters in fall of 2009 (Figure 4, top panel).

Ms signals were very rare in SOCAL, but appeared at site M only in July and September of 2010 over the three year monitoring period. Also, two days with Ms acoustic encounters occurred over a one week monitoring period at a site off the shelf of Southern California (33°28.4′N, 122°31.4′W, CCE1, Figure 2) in late spring of 2009, but these were discarded from the comparative analysis due to brief effort. During 2010 at the Aleutian Island site Buldir, Ms signals were detected prior to November and after December. There were a high number of Ms signal encounters offshore of the Washington coast in the first six months of 2011, with a sudden drop in July and no further encounters throughout October of 2011. Acoustic encounters of Ms type in the Gulf of Alaska occurred with a peak in August 2011 and a gradual decrease until February 2012 (Figure 4, middle panel).

Acoustic encounters of Md signals occurred mainly around the Pacific Islands sites. Noteworthy were the higher numbers of acoustic encounters over the late spring and early summer months at the island of Hawai’i with few or no detections over late fall and winter between November and March. From one year of recordings from Kaua’i, there were many Md acoustic encounters observed in the fall of 2009 followed by lower encounter rates throughout the rest of the recording year (Figure 4, bottom panel).

### Diel Pattern

**Figure 5:**
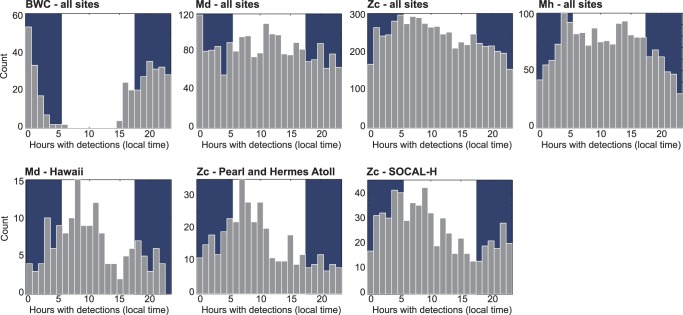
Diel cycle of Cross beaked whale (BWC), *M. densirostris* (Md), *Z. cavirostris* (Zc), and *M. hotaula* (Mh) pooled over all sites and geographic regions (top panel), as well as for Md and Zc at select sites. Dark shaded areas indicate nighttime. BWC displayed a clear nocturnal activity over all sites, Mh had a diel pattern with higher acoustic activity during the day, Zc had a slight diel pattern with higher activity from after midnight to mid-day over all sites, particularly pronounced at Pearl and Hermes Reef and SOCAL H. Md did not have a diel pattern, except at Hawai’i, where the pattern was similar to Zc.

The BWC FM pulse type was the only signal that was recorded during only one portion of the day, with most acoustic activity at night across all sites (Figure 5, top left). The Mh signals, dominated by large number of detections at Kingman Reef, had a diel pattern with higher acoustic encounter rates during the day. When sites with encounters of Zc signals were pooled, this FM pulse type displayed a trend of higher acoustic activity between midnight and midday and lower activity in the afternoon and first half of the night (Figure 5, top 2^nd^ from right). This pattern was particularly pronounced at Pearl and Hermes Reef, and SOCAL H (Figure 5, bottom middle and right), but also at SOCAL G2, and N. SOCAL M had an opposing pattern with highest activity in the afternoon. Most other sites did not have enough data with Zc signals or did not display a diel pattern. Md signals pooled across all sites did not show a diel pattern (Figure 5, top center). However, at Hawai’i (Figure 5, bottom left), and to a lesser extent at Kaua’i, the diel pattern for Md signals was similar to what was observed for Zc signals with highest acoustic activity in the early morning hours to midday.

## Discussion

### Acoustic Analysis

Manual detection of FM pulse type acoustic encounters typically provides a low number of missed or false detections. The method of screening long-term data with LTSAs offers a fast overview for long periods of time (usually 1 h) and allows for efficient data analysis. Despite the compressed view, the analyst is capable of detecting periods with very few (∼2) echolocation signals. However, we have found that results can vary even between experienced analysts, particularly in data sets with many different species emitting echolocation signals simultaneously. Therefore, while the number of missed detections is relatively low, precise characterization remains elusive. A multi-step labeling process minimized the number of false classifications and we are confident that these labels and categories are valid.

Beaked whale FM pulses have been proven to be species-specific for a number of described species with one FM pulse type per species [8]–[10], [13], [14], [22]. Besides FM pulses, some beaked whales also produce dolphin-like echolocation clicks and dolphin-like buzz clicks [9]–[12]. It cannot be ruled out that a beaked whale species may produce multiple FM pulse types, possibly with geographic variation; however, such behavior has not been reported from acoustic recordings with visual confirmation. Also, while there may be geographic variation, which has yet to be quantified, FM pulse characteristics seem to be stable enough across ocean basins to reliably categorize FM pulse types to known species (SBP unpublished data) in well-known species like Zc.

Echolocation signals of odontocetes are known to vary largely in their frequency content based on the orientation of the animal in relation to the recording hydrophone due to its highly directional echolocation beam [23]. Additionally, higher frequencies attenuate more over distance than lower frequencies such that distance of the animal to the hydrophone will impact the frequency content of the received signal. However, in a situation where similar issues are expected, Soldevilla et al. [21] have shown consistent spectral patterns on long-term acoustic recorders for two dolphin species. While there is considerable variability within each species, the overall shape of the spectra from the onset of the broadband energy up to the peak frequency and possible smaller spectral peaks and notches below the main energy appear to be consistently recurring patterns. Frequencies beyond the peak are highly variable, likely depending on the angle to and distance between the animal and the hydrophone.

Besides the FM pulse types of known species, we describe the spatio-temporal distribution of four FM pulse types (BWC, BW40, BW43, BW70), for which visual identification of the species producing these signals do not yet exist [5], [15] (Figure 1).

The categories were manually identified based on spectral and temporal grouping. It has yet to be shown if these signals are indeed species-specific and produced by beaked whales. The BW40 signal type is very similar to Zc signals, but mostly occurred with dolphin-like echolocation clicks in a continuous sequence, whereas Zc is not known to make dolphin-like clicks aside from buzz sequences. It is possible that, based on the similarity to Zc signals and the high relative presence of Zc signals in the region, this signal type was more frequent and misclassified as Zc, or that Zc produces other signal types under some conditions. However, while the main frequency content of the BW40 FM pulse is highly similar to the Zc signal, the two spectral peaks at 17 and 23 kHz common in Zc signals were not present in the BW40 encounters. Additionally, while not all BW40 pulses show a long duration, the signal duration of most BW40 FM pulses were distinctly longer than what is usually found for Zc signals (Figure 1).

Manual detection of the Ip pulse type has proven difficult and conclusive long-term and spatial results have yet to be obtained for this signal type. While we did have few Ip signal encounters, at Palmyra Atoll and at Pearl and Hermes Reef, we are likely to have missed a number of acoustic encounters. Ip has three types of echolocation signals, an FM pulse with 25 kHz peak frequency, and two dolphin-like echolocation clicks with 15 and 25 kHz peak frequency [10]. Current knowledge suggests that only about one third of Ip signals are FM pulses and the time spent during manual analysis, browsing signals in each sequence for FM pulses, may not be sufficient to classify these few encounters as Ip, with an analyst instead labeling these signals as belonging to other odontocetes.

### Relative Presence

Relative site presence might be seasonally dependent, such that our results may be biased for those sites where there was only a partial year of data. An example of this is the Aleutian Islands Kiska (KS) site, which had only 48 days of data recorded during the late spring and early summer of 2010. While 25% of the Ms signals were detected at Kiska, Aleutian Islands site Buldir (BD) reports only 5% of the detections. However, the BD deployment covers 272 days and Ms signals were not detected in the months of November and December, lowering the contribution. While there are many sites with multi-season and even multi-year effort, there are enough sites with shorter monitoring periods that caution should be used to not over-interpret the results.

Relative site presence also should not be directly related to abundance. Based on observed source levels for Zc and Md, the expected detection range is no more than several km [24]. Detection range will vary with species source level, echolocation beam pattern, and hydrophone placement. In addition, Blainville’s and Cuvier’s beaked whales exhibit some degree of spatial niche separation around Hawai’i [25]. Consequently, variations in relative site presence may be expected with these species and possibly others when recording at a nearby site.

Another source of variation in the relative occurrence of FM signals among sites is shown from the results at the two very closely located Palmyra Atoll sites and over the larger SOCAL region. While both sites at Palmyra Atoll were in similar water depths and there were no apparent reasons why one site should be favorable to the other, there was a higher site presence at the northern shore (NS) site compared to the southern (WT) site. Prey aggregation based on oceanographic factors favoring the north shore of the atoll may have caused this difference. Assessment of relative occurrence in a particular region may be strongly impacted by the choice of monitoring location, such that inferences on the presence or absence of a species based on a single site recording should consider the potential for local oceanographic or other habitat variables, which may influence the ability to detect a species. Comparison of FM signal encounters across the entirety of the Southern California Bight, based on several recording sites, likely provides a good assessment of overall beaked whale occurrence and how habitat preferences influence relative distribution. The lack of acoustic encounters of FM pulse types on three additional southern California sites that were omitted from analysis (SOCAL A, B, and G) is likely related to their shallow deployment depths between 300 and 600 m, shallower than is expected for beaked whale habitat [26]. Also, the Washington Cape Elizabeth site with a water depth of 100 m was an unlikely site for beaked whales but it had a few encounters of Bb FM pulse types. SOCAL C was dominated by Bb FM pulse encounters with only few acoustic encounters of BW40 signals. SOCAL C is located at 800 m depth (Table 1) on a slowly down sloping area on the shelf at the entrance to the Santa Barbara Basin. It would be interesting to investigate whether Bb preferred this type of bathymetric and topographic feature over the steep slopes that Zc favor [26].

### Seasonal and Diel Pattern

Seasonal patterns of beaked whale presence throughout the study area were small, inconsistent, or lacking in data. Ms signals were detected prior to November and after December at the Aleutian Islands site Buldir (BD). Fewer winter acoustic encounters indicate that the species producing this signal, likely *M. stejnegeri* [14], may not completely leave the area for the entire winter season. Conversely, at the Gulf of Alaska and Washington Quinault Canyon (QC) sites, Ms signals occurred throughout the entire winter with a sudden disappearance of acoustic encounters at QC in the summer from July to the end of the recording period, October of 2011 (Figure 4, middle panel). This might suggest seasonal latitudinal movement with the use of more northerly sites during summer months.

It is tempting to infer seasonal movement of Md between Hawai’i and Kaua’i based on the pattern of occurrence within those datasets; however, large gaps in each dataset and analysis of only a single year from each site suggest additional data are needed before such an assertion can be supported. The movement patterns of many insular Hawaiian odontocetes [27], [28] are characterized by periods of short-term residency within a relatively small area, followed by long-distance movements to other locations. These patterns are likely driven by corresponding changes in the distribution of their prey.

A strong diel pattern was only observed for BWC FM pulses. Since beaked whales emit FM pulses predominantly during foraging dives [6], [7], the diel pattern of echolocating indicates a foraging strategy different to that of other beaked whale species and is possibly related to the behavior of the preferred prey species. Blainville’s and Cuvier’s beaked whales are known to echolocate at depths between 200 and 1900 m, but most echolocation activity occurs below 450 m for both species [4], [6]. The recording site at Cross Seamount was located at less than 400 m water depth on the top of the seamount. Assuming for this species a similar dive and echolocation behavior as Blainville’s and Cuvier’s beaked whales, as well as considering the highly directional beam pattern common to all currently known echolocating odontocetes, Johnston et al. [29] hypothesized that the BWC diel pattern occurred not due to primarily nocturnal foraging but due to vertical prey movement in the water column, which allowed foraging at depth during the day, beyond the detection range of the recorder, and in shallower water near the recorder during the night. The HARPs at Kaua’i, Pearl and Hermes Reef, and Wake Atoll, all at depths of 700–800 m, recorded the same diel pattern for BWC FM pulses. Therefore it is plausible that the observed diel pattern is due to nocturnal foraging rather than an artifact of the hydrophone depth.

The variability of diel patterns, or the lack thereof depending on location, for Mh, Zc and Md signals shows a very different foraging strategy than the species producing BWC signals. The regional differences likely represent the type of prey sources particular to the site, or differing foraging strategies among regional populations. A comparison of stable isotope ratios from biopsy samples on a regional scale might shed light on these differences.

### Geographic Distribution of Beaked Whale Species and FM Pulse Type

Beaked whale stranding or sighting records have been summarized by MacLeod et al. [30]. In the following discussion we report the first record of each beaked whale species in the vicinity of our acoustic recorder locations. This gives the opportunity to confirm the distribution of species with known FM pulse types and to link the unknown FM pulse types based on their geographic distribution with potential beaked whale species in that area.

#### Berardius bairdii – produces Bb FM pulse

Baird’s beaked whale is found in cold-temperate waters of the North Pacific, like M. stejnegeri and M. carlhubbsi, but based on strandings and sightings it has a larger range than either of the other two species and is more abundant [31]. In the eastern part of their range, strandings are not common, but they are known from various locations in Alaska and south to British Columbia, Washington, California [32], [33], and Baja California, Mexico [30]. Near the southernmost part of Baja California, Mexico, in the Gulf of California, Baird’s beaked whales are known from three mass strandings, two near La Paz in July 1986 and one from Isla San Jose in July 2006 [34]. In the western North Pacific, Baird’s beaked whale strandings are known from Commander Islands, Russia; Kamchatka, Russia; and Japan [35]. The southernmost record in the western Pacific is from China in the East China Sea approximately 30°N; no records exist from Taiwan [36]. This species is not known nor expected from the regions around any of our tropical recording sites (Northern Line Islands, Hawai’i, Kaua’i, Pearl and Hermes Reef, Wake Atoll, and Saipan).

**Figure 6:**
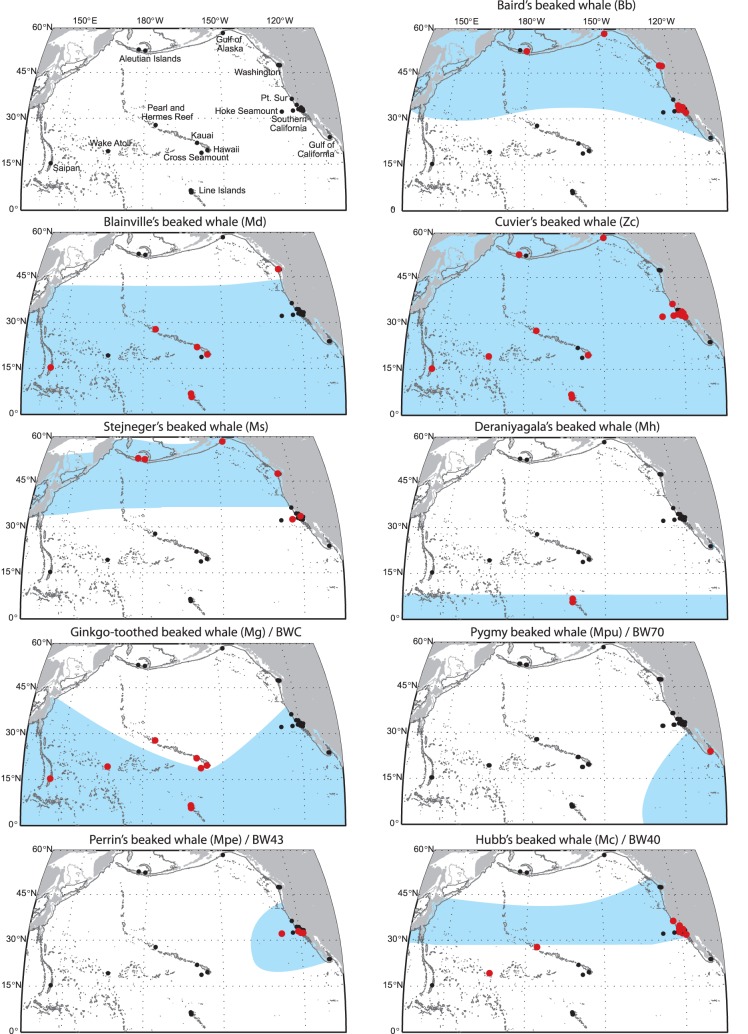
Distribution maps (light blue shaded area; adapted from [102]) of all known beaked whale species in the North Pacific (except Ip). Acoustic encounter locations of title species (red circles) of known FM pulse types (Bb, Md, Zc, Ms, Mh) or potential FM pulse matches (Mg/BWC, Mpu/BW70, Mpe/BW43, Mc/BW40). Location of HARPs with no acoustic encounter of title species (black circles).

Acoustic recordings confirmed the distribution of Baird’s beaked whales in cold-temperate waters with acoustic encounters around the Aleutian Islands, Gulf of Alaska, Washington, and Southern California (Table 4, Figure 6). Baird’s beaked whale was the second most frequently acoustically encountered species in these regions after Ms or Zc, respectively.

**Table 4:** Presence (+) or absence (−) of beaked whales based on strandings (S), visual sightings (V), and acoustic FM pulse encounter (A). HI: Hawaiian Islands.

	Bb			Md			Zc			Ms			Mh			Mg		BWC	Mpu		BW70	Mpe		BW43	Mc		BW40
Project Area	S	V	A	S	V	A	S	V	A	S	V	A	S	V	A	S	V	A	S	V	A	S	V	A	S	V	A
Aleutians	+	−	+	−	−	−	+	+	+	+	+	+	−	−	−	−	−	−	−	−	−	−	−	−	−	−	−
Gulf of Alaska	−	−	+	−	−	−	+	−	+	−	−	+	−	−	−	−	−	−	−	−	−	−	−	−	−	−	−
Washington	+	+	+	−	−	+	+	−	−	+	−	+	−	−	−	−	−	−	−	−	−	−	−	−	+	**−**	**−**
Point Sur	+	+	−	−	−	−	+	+	+	+	−	−	−	−	−	−	−	−	+	−	−	−	−	−	+	−	+
SOCAL	−	+	+	+	−	−	+	+	+	+	−	+	−	−	−	+	−	−	+	−	−	+	−	+	+	−	+
Gulf of California	+	−	−	−	−	−	+	+	−	−	−	−	−	−	−	−	−	−	+	+	+	−	−	−	−	−	−
Main HI	−	−	−	+	+	+	+	+	+	−	−	−	−	−	−	−	−	+	−	−	−	−	−	−	−	−	−
NW HI	−	−	−	+	−	+	+	+	+	−	−	−	−	−	−	−	−	+	−	−	−	−	−	−	−	−	+
Northern Line Islands	−	−	−	+	−	+	+	+	+	−	−	−	+	+	+	−	−	+	−	−	−	−	−	−	−	−	−
Wake Atoll	−	−	−	−	−	+	+	−	+	−	−	−	−	−	−	−	−	+	−	−	−	−	−	−	−	−	+
Saipan	−	−	−	−	−	+	+	−	+	−	−	−	−	−	−	−	−	+	−	−	−	−	−	−	−	−	−

#### Mesoplodon densirostris – produces Md FM pulse

Blainville’s beaked whale has the most wide-spread and varied distribution of any Mesoplodon species. It is found in tropical and warm-temperate waters, including offshore, deep waters, around tropical oceanic archipelagos, and on continental or insular coasts of these areas. M. densirostris is rarely sighted, and field identification is difficult. Its distribution has been inferred mainly from stranding records.

In the eastern North Pacific, sightings of Md are rare off California [37] to waters offshore of Costa Rica [38] and southward. In the central North Pacific, sightings and strandings occur from the Hawaiian Islands (Oahu, Hawai’i, Molokai, Kaua’i, and Laysan), the Society Islands, the Line Islands, and Midway Atoll. Strandings have occurred in the western North Pacific in Japan (Ryukyu Islands, Kyushu, and Honshu), the Philippines, and Taiwan [39], [40].

Strandings or sightings of *M*. *densirostris* at or near our recording sites include the following: Point Sur (Pescadero Beach, CA) [41]; Palmyra Atoll [42]; Hawai’i (sightings) [43]; Pearl and Hermes Reef (from Laysan) [44]. There have been no strandings or sightings of this species from the Aleutian Island chain, Alaska or British Columbia, Canada Washington, or from the cold-temperate region of the western North Pacific [35], nor are they expected from these regions.

Md FM pulse type encounters have higher encounter rates in subtropical to tropical regions, with Pearl and Hermes Reef, Saipan and Hawai’i having the greatest percentage of days with detections, confirming what is known about *M. densirostris* preferred range (Table 4, Figure 6). All Pacific Islands sites except Cross Seamount and Wake Atoll had Md FM pulse type encounters. The only other site with a single Md signal encounter, outside the Pacific Islands region, was off the coast of Washington.

#### Ziphius cavirostris – produces Zc FM pulse

Cuvier’s beaked whales occur in deep waters worldwide, both nearshore around oceanic islands and in the open ocean, and ranging from equatorial tropical to cold-temperate waters. However, they are not reported from high latitude polar waters [45], [46].

Cuvier’s beaked whales are perhaps the most common of all beaked whales, with more reports of sightings and strandings than any other ziphiid species [46]. Where steep slopes occur close to shore, such as around the Hawaiian Islands and San Clemente Island, offshore of southern California, their regular appearance allows for photo-identification and tagging studies [43], [47]. As for other beaked whale island populations, they appear to be at least seasonal residents. Around Hawai’i, the re-sightings of identifiable individual Cuvier’s beaked whales span over 15 years and suggest they are resident with long-term site fidelity [43].

Cuvier’s beaked whales are the most common beaked whales to strand throughout the North Pacific rim and on islands. As a comprehensive list, starting in Alaska and moving clockwise around the North Pacific, Zc strandings are known from the Aleutian Islands (Samalga Island), USA [48]; Bella Bella, British Columbia, Canada [49], [50]; North Ocean Lake, Washington [49], [51]; Del Mar, California [49], [52], [53]; San Ramon, Baja California Norte, Mexico [49], [54]; and Gulf of California, Mexico [49], [55]. In the western North Pacific counterclockwise from Alaska, Zc strandings are known from Bering Island, Commander Islands, Russia [56]; off Korean Peninsula (bycatch) [57]; Miura City, Japan (RO-079, ICR); Lukang, Taiwan [58]; China [36]; Philippines (sighting) [59]. On the offshore and oceanic islands, Zc strandings are known from Guam (RLB unpublished record), Wake Island [41]; Sydney Island, Phoenix Islands, Kiribati [60]; Pohnpei, Caroline Islands [60]; Midway Atoll [61]; Kalae, Hawai’i [62]; Johnston Island [63]; and Palmyra Atoll [60].

Comparing sighting and stranding data to their acoustic encounters, confirms that Zc signals are the most commonly heard FM pulse type and with the broadest geographic range (Table 4, Figure 6). The only large regions in the North Pacific where no Zc signals were encountered were off the coast of Washington, and the Gulf of California. However, Barlow et al. [31] reported that the highest density of Zc in the Pacific is in the southwest Gulf of California. This discrepancy can likely be explained by an undersampling of the Gulf of California, the choice of recorder location covering only a very small portion of suitable habitat, and the pattern of a species dominating each site described within this paper.

#### Mesoplodon stejnegeri – likely produces Ms FM pulse

Stejneger’s beaked whale was first described in 1885 from a skull collected on Bering Island, Commander Islands, Russia [64]. This species has not been reported from any central Pacific islands. By the late 1980s, there are 48 records of this species from the North Pacific [41]. Four mass strandings of this species have been reported from Kuluk Bay, Adak, Alaska between 1975 and 1989 [65]. Other Bering Sea stranding locations include: Shemya Island, Amchitka Island, Adak Island, Saint Paul Island, and Tanaga Island. There are numerous additional stranding records for this species in the cold-temperate waters of northern Japan [66]. The southernmost stranding from the California Current was at southern Cardiff-by-the-Sea, California [66]. The southernmost stranding of this species was near the southern front of the Japanese cold water Oyashio Current at Tsuyazaki, Fukuoka south of Tokyo [66]. Therefore, the southern limit is about the same latitude on both sides of the Pacific. In the Sea of Japan, a single specimen was reported in market samples of cetacean products from Korean “whale meat” markets [57] and a few strandings are known from the Korean Peninsula [67].

Strandings of *M. stejnegeri* at or near our recording sites include the following: Aleutian Islands (mass stranding events of this species are known from Adak, Tanaga, Shemya, and Unalaska Islands) [14]; Washington (Leadbetter Point, Waatch River, and Twin Harbors State Park) [41]. Stejneger’s beaked whales are known as cold-temperate species. They are not known from the regions around any of our subtropical or tropical recording sites (Gulf of California, Palmyra Atoll, Hawai’i, Kaua’i, Pearl and Hermes Reef, Wake Atoll, and Saipan).

Acoustic encounters of the Ms FM pulse type dominate Aleutian sites, Gulf of Alaska, and the offshore Washington site, strengthening the hypothesis that this signal type is produced by *M. stejnegeri.* Also, this signal type was on rare occasions found on some of the SOCAL recordings confirming the species’ range known from sightings and strandings (Table 4, Figure 6).

#### Mesoplodon hotaula – produces Mh FM pulse

Deraniyagala’s beaked whale is known from only seven confirmed specimens [3]. These are: (1) the holotype, from Ratmalana, Sri Lanka, (2) Tabiteuea Atoll, Kiribati, (3–5) Palmyra Atoll, Northern Line Islands; (6) Hulhudhuffaaru, Raa Atoll, Maldives; and (7) Desroches Island, Seychelles. These beaked whales are best known from Palmyra Atoll, Northern Line Islands (05°50′N, 162°06′W) where three specimens have stranded and where live animals have been observed around the atoll [13].

No Deraniyagala’s beaked whale type signals were recorded from any site other than the Northern Line Islands. Because this species appears to be restricted to tropical waters, it would not be expected near the Aleutian Islands or in the temperate eastern Pacific. To date, no specimen or sighting of *M. hotaula* is known east of the Line Islands. There are also no specimens or possible sightings from any of the Hawaiian Islands. The Mh FM pulse type was only found at Palmyra Atoll and Kingman Reef and was the most common beaked whale species at both locations (Table 4, Figure 6). Kingman Reef had the highest relative presence (100%) for beaked whales of all sites, and 78% of the encounters were with Mh. Within the regions that were acoustically monitored, the only sites outside the Northern Line Islands where the Mh signal type might be expected would be near Saipan and Wake Atoll, however none have been identified to date at those sites.

#### Indopacetus pacificus – produces Ip FM pulse

Longman’s beaked whale is another poorly known monotypic beaked whale occurring in the southern part of the North Pacific and into the warm-temperate and tropical waters of the South Pacific, and westward into the tropical northern and central Indian Ocean. The first stranding was collected in northern Australia at Mackay, Queensland in 1882 [68]. The next specimen was collected near Danane, Somalia in 1956 [69]. Dalebout et al. [70] reported on four new specimens from the western and central Indian Ocean. Over the past ten years only eight more specimens have been identified and these were found in the Maldives, Myanmar, Philippines, Taiwan, and Japan [71]. There were also sightings of these whales in the Eastern Tropical Pacific [30].

The only known stranding from the Central North Pacific is a recent specimen from Hawai‘i [72]. As Longman’s beaked whale is only known from tropical waters in the Indian and Pacific Oceans, they are not known from strandings or sightings from the regions around any of our cold-temperate water recording sites (Aleutian Islands, Washington, Pt. Sur, and Southern California).

There were too few acoustic encounters of Ip FM pulse types to be included in the quantitative analysis. The few positively identified acoustic encounters were from Palmyra Atoll and Pearl and Hermes Reef, which fall into the expected distribution for this species (Table 4, Figure 6).

#### Mesoplodon ginkgodens – possibly produces BWC FM pulse

Ginkgo-toothed beaked whales are found in warm-temperate and tropical waters of the Pacific and westward into the Indian Ocean to at least the Maldives [73]. This species was first described from Japan, based on a specimen from Oiso Beach, Sagami Bay, Japan [74]. Based on strandings or capture records, this species is most common around Japan and also reported around Taiwan [75]. Specimens are also known from Liaoning Province, China [76]; Del Mar, California [77]; a specimen previously identified as M. ginkgodens from Baja California, Mexico [78]–[80] has recently been reidentified as M. peruvianus [81]; Galapagos Islands [82]; Strait of Malacca, Indonesia [41]. In the Southern Hemisphere, individuals have stranded in southeastern New South Wales, Australia [83] (reported as M. bowdoini), [84], and Bay of Plenty, New Zealand [85]. The specimen of M. ginkgodens from Chatham Islands [86] has been reidentified as M. grayi [85]. However, the identification of some of these specimens is in question [3]. Strandings of M. ginkgodens are not common anywhere, but the largest number of records are from Japan; however, there are no confirmed strandings of this species at or near any of our recording sites. A specimen taken near Pohnpei (06°50′N, 158°15′E) was mistakenly first reported from Guam [29] is the closest confirmed M. ginkgodens record to Palmyra Atoll. The range of M. ginkgodens in the central Pacific is poorly known and may or may not overlap with M. hotaula, but specimens of both species have not been reported from near-by areas, but BWC FM pulse types recorded off Kingman Reef and Palmya Atoll support the idea that these two species are occasionally sympatric in the central Pacific.

The properties of echolocation signals produced by *M. ginkgodens* are unknown. However, its distribution overlaps with the occurrence of the BWC FM pulse type (Table 4, Figure 6). The BWC signal was encountered on all Pacific Islands sites, dominating Cross Seamount detections and contributing to a large part of Saipan detections. They were not heard in any of the other regions.

#### Mesoplodon peruvianus – possibly produces BW70 FM pulse

Stranding and sighting records suggest that pygmy beaked whales are primarily found in tropical and warm-temperate waters of the eastern Pacific [81]. The northernmost record of this species was a specimen that stranded alive in Moss Landing, California, in January 2001. Another, specimen stranded at Newport Beach, California, in February 1998. The northernmost sighting of this species at-sea was from 26° 10′N 110° 48′W on 11 August 2006, in the central Gulf of California, Mexico (NOAA Southwest Fisheries Science Center unpublished data). The southernmost record in the eastern Pacific was a stranded specimen from northern Chile (Punta de Choros), collected in May 1995 [87]. The only record of this species away from the eastern Pacific was a stranding of a 327 cm male from Oaro, Kaikoura, South Island, New Zealand in 1991 [85]. Whether this specimen is indicative of a wider distribution for this species, or just an errant individual is unknown, but it seems unlikely this species would normally occur in the cooler waters around New Zealand. In addition, New Zealand has the oldest stranding program in the Pacific (from the 1860s) but only the 1991 specimen has been identified as M. peruvianus.

Based on 24 at-sea sightings (of *Mesoplodon* sp. A) presented by Pitman et al. [88] and 85 different sightings by Hamilton et al. [89], *M. peruvianus* appears to be endemic to the eastern tropical Pacific Ocean. Most at-sea sightings have been concentrated in the warmest waters of the ETP, the “Eastern Pacific Warm Pool”, an area with sea surface temperatures >27.5°C [90]. Comparing the plots in Fig. 27 (*M. peruvianus*) with Fig. 28 (*Mesoplodon* sp.) in Hamilton et al. [89] it seems likely that this species may be particularly abundant in the southern Gulf of California (see also [91]). Also based on all records, it seems unlikely that this species would have been recorded from our other recording sites in the Central and Western Pacific if they were truly endemic to the warmest waters of the ETP.

While no acoustic recordings have been collected in the presence of Mpu beaked whales, the most likely FM pulse type to fit the distribution of this species would be the BW70 signal, recorded only in the Gulf of California (Table 4, Figure 6) at the core of the species′ habitat, and with *Z. cavirostris* expected to be the only other beaked whale species found there.

#### Mesoplodon perrini – possibly produces BW43 FM pulse

Perrin′s beaked whale is known only from five strandings along the coast of southern California [92]. The species is apparently rare, as there have been no confirmed sightings during cetacean abundance surveys conducted by NOAA in Californian waters. Based on the limited region of known strandings, this species appears to have the most restricted range of any species of Mesoplodon, occurring in the warm-temperate waters off southern California (this region is the border between the cold-temperate waters to the north and the warm-temperate waters to the south) and likely at least offshore of northern Baja California, Mexico, as well [93].

Echolocation signal properties for *M. perrini* are unknown. Given its restricted range, the BW43 FM pulse type seems to fit this distribution very well (Table 4, Figure 6). BW43 signals were detected at deep sites (1100–1300 m, Table 1) at the southwestern edge of the Southern California Bight (sites E and N), at the shelf break (site SN), and offshore at Hoke Seamount west of San Diego, California.

#### Mesoplodon carlhubbsi – possibly produces BW40 FM pulse

Hubb′s beaked whale is one of two Mesoplodon species, the other being M. stejnegeri, endemic to the cold-temperate waters of the North Pacific. However, Hubb’s beaked whales found in the cold water of the Oyashio Current off northern Japan were believed to be disjunct from those living in the cold-water California Current [35], [94]. The southernmost stranding in Japan is from Numazu, Suruga Bay [95] and the northernmost record is from Ayukawa [96]. There are no records from Korea as strandings or bycatch from the Sea of Japan [57], [97]. There are no strandings of this species from the Aleutian Chain, Alaska, or the Hawaiian Islands [35], [94]. In the eastern North Pacific, the northernmost stranding is from Prince Rupert, British Columbia [98], [99] and the southernmost stranding is from San Diego, California [53], [94]. MacLeod et al. [30] speculated that the two North Pacific populations might be continuous across the North Pacific around 30°N and 45°N. This idea is supported by a specimen of Mc collected by an observer from the middle of the North Pacific [100] while on board a fishing vessel operating in the High Seas Driftnet fisheries (conducted by Japan, Korea, and Taiwan) in the central North Pacific.

Strandings of *M. carlhubbsi* at or near our recording sites include the following: Washington [100]; Pt. Sur (Cypress Point and San Simeon Bay) [94]; central California and San Clemente Island [100]; Hubb’s beaked whales are a well-known cold-temperate species and they are not known from the regions around any of our tropical recording sites (Gulf of California, Palmyra Atoll, Hawai’i, Kaua’i, Pearl and Hermes Reef, Wake Atoll, and Saipan). Based on the known distribution of this species it is also not expected to occur around the Aleutian Islands.

Hubb’s beaked whale echolocation signals have been recorded and described from two young, possibly neonate, and stranded animals under emergency care in captivity [101], [102]. The described signals were either recorded with a sampling rate lower than necessary to likely describe the full bandwidth of the echolocation signal of beaked whales [102], or presented in a way that the typical beaked whale FM pulse properties – sweep and inter-pulse interval – were not clearly identifiable and signal parameters were not comparable to this study.

Hubb’s beaked whale may produce the BW40 FM pulse type. Acoustic encounters of BW40 at central and southern California sites correspond well with the expected range (Table 4, Figure 6). Lack of detections in the northern range and positive detections at Pacific Islands sites are unexpected and weaken the assumption that the BW40 type is indeed linked with Hubb’s beaked whale echolocation signals.

## Conclusions

Passive acoustic monitoring of elusive beaked whales has proven to be a feasible technique to study the distribution and relative presence of these species throughout the North Pacific. Comparison with sighting and stranding data, passive acoustic monitoring has provided a good indication for probable species producing previously undescribed beaked whale-like signals. Relatively low numbers of acoustic encounters with rare signal types, the lack of multi-year data for many sites, variability within a region, and low density of recording instruments reduced the value for interpreting seasonal movement and diel foraging patterns. However, with continuing data collection this caveat can be reduced. Furthermore, knowledge gained about behavioral and distributional patterns of rarely observed species may help with planning fieldwork for concurrent acoustic and visual species identification. Future research should investigate how habitat preference and local oceanographic features rather than large-scale seasonal aspects may control prey abundance and in return beaked whale presence, particularly at temperate and tropical sites.
