# Correction: Spatio-Temporal Patterns of Beaked Whale Echolocation Signals in the North Pacific

**DOI:** 10.1371/journal.pone.0091383

**Published:** 2014-03-07

**Authors:** The PLOS ONE Staff

There are errors in the Funding section. The correct Funding information is as follows:

Funding provided by United States Office of Naval Research, M. Weise (http://www.onr.navy.mil/en/Science-Technology/Departments/Code-32/All-Programs/Atmosphere-Research-322/Marine-Mammals-Biology.aspx), United States Navy Living Marine Resources, B. Gisiner and F. Stone (http://www.lmr.navy.mil), United States Pacific Fleet, C. Johnson (https://portal.navfac.navy.mil), Pacific Life Foundation, B. Haskell (http://www.pacificlife.com/PL/FoundationCommunity/Overview/Corp_PLF_Home.htm), Ocean Foundation, M. Spaulding (http://www.oceanfdn.org), Naval Postgraduate School, C. Collins and J. Joseph (http://www.nps.edu), National Oceanographic Partnership Program (http://www.nopp.org), the Bureau of Ocean Energy Management, J. Lewandowski and J. Price (http://www.boem.gov), Pacific Islands Fisheries Science Center (PIFSC), National Oceanic and Atmospheric Administration (http://www.pifsc.noaa.gov/cetacean). The funders (except for PIFSC, EMO) had no role in study design, data collection and analysis, decision to publish, or preparation of the manuscript.
